# Numerical Investigation of a Mitochondria-Inspired Micromixer for Enhanced Mixing

**DOI:** 10.3390/mi17050525

**Published:** 2026-04-25

**Authors:** Muhammad Ali Hashmi, Arvydas Palevicius, Sigita Urbaite, Giedrius Janusas, Muhammad Waqas

**Affiliations:** Faculty of Mechanical Engineering and Design, Kaunas University of Technology, 51424 Kaunas, Lithuania; sigita.urbaite@ktu.lt (S.U.);

**Keywords:** computational fluid dynamics (CFD), bio-inspired, microfluidics, mitochondria, chaotic advection, passive mixing

## Abstract

Today, microfluidics has become a revolutionary field of engineering due to its wide range of applications, including lab-on-a-chip devices, microscale biochemical reactors, drug delivery systems, and disease diagnostics. Efficient fluid mixing has been a significant challenge in these systems due to the dominance of laminar flow and low-Reynolds number conditions, where mixing relies primarily on slow molecular diffusion. It is very difficult to achieve rapid mixing and homogeneous mixing within a limited length. In this study, a bioinspired passive micromixer is developed based on the cristae architecture of mitochondria, which is known for maximizing surface area and transport efficiency in biological systems. The micromixer incorporates cristae-like microstructures within a straight microchannel to produce continuous flow deflection, stretching, and folding, thereby promoting chaotic advection without relying on external energy sources. It also includes mitochondrial granules, such as micropillars, within the channel to disrupt streamline flow. Thus, a numerical investigation was conducted to design four different micromixer geometries: conventional T-channel, and T-channels with a single, double and triple matrix of cristae. The analysis was performed in COMSOL Multiphysics, in which “Laminar flow” and “Transport of diluted species” physics were used, and a stationary study was executed. Simulations were conducted at different Reynolds numbers (Re = 0.1–100) to observe the feasibility of the proposed designs. For analysis, the mixing index and concentration profiles at the outlet and along the length were also examined. The results showed that the high cristae density channel performed well, achieving a mixing index of 95.85% at Re = 0.1 and 85.84% at Re = 100, proving that the proposed mitochondria-inspired cristae Mito-mixer delivers efficient mixing over a broad Reynolds-number range while maintaining a compact, length-efficient design.

## 1. Introduction

Recent developments in microfluidics and lab-on-a-chip devices have revolutionized several biomedical applications, including chemical analysis, by enabling fluid manipulation in response to user needs. The need for the effective mixing of particles at low Reynolds numbers is observed in various applications, such as drug delivery systems, biochemical reactors, etc. [1]. For example, a multi-inlet vortex mixer (MIVM) is used for the green synthesis of nanosilica. Nevertheless, significant challenges have been faced when trying to manipulate fluids for mixing [2]. At low Reynolds numbers, the fluid particles follow the same path without interfering with other particles. In this case, viscous forces dominate the inertial forces. The biggest challenge is when the Reynolds number becomes very low, and the laminar flow starts behaving like creep flow. At the macroscale level, inertial forces dominate the viscous forces, helping the fluid create some turbulence and favoring mixing. Due to a lack of this trait at the microlevel, the mixing of fluid takes place by diffusion only, and it requires long channels to do so [3]. Typically, T- and Y-mixers are used under normal conditions to allow fluids to diffuse along long channels and mix. It is a very slow process, motivating researchers to provide better solutions [4]. To overcome this limitation, many researchers have tried various approaches to achieve the desired results with maximum efficiency. Many of them have focused on inducing chaotic advection. Chaotic advection is a process in which fluids are manipulated in complex channels, thereby enhancing the contact area between particles. Different researchers have employed a variety of approaches to achieve this effect [5].

According to dynamic system theory, chaotic particle motion may occur when a velocity field is either two-dimensional and time-dependent or three-dimensional. To induce chaotic mixing in the channel, twisted microchannels with inclination, oblique and wavelike structures were designed. This helped in decreasing the mixing length by up to 31% from the basic T-mixer [6]. Introduction of the Archimedes screw in a Y-shaped channel increases not only chaotic advection, but also the mixing efficiency when compared to typical Y-shaped mixers [7]. Thus, a device was developed that combined the zigzag geometry with gear-shaped lateral walls and bullet-shaped flow obstacles to extensively increase chaotic advection [8]. As later research showed, a C-shaped multidirectional micromixer design with a radial baffle across the channel also provided good mixing [9]. A rectangular sinusoidal channel was used for rapid and low-cost PCR (polymerase chain reaction) development and the numerical study of micromixer effects [10]. Koch snowflake fractal baffles (KSFB) also increased the chaotic advection and mixing efficiency [11]. The Split and recombine (SAR) strategy guides the flow to collide, inducing chaotic advection. This method attracted some researchers and seemed to be useful in mixing at this level [12]. This principle was also used to develop a micromixer device, which was very efficient for protein phosphorylation in human embryonic kidney cells (HEK) upon epidermal growth factor (EGF) [13]. A novel Tesla micromixer [TSM] was designed by introducing an asymmetric channel arrangement. The results indicated that fluids of varying concentrations were continuously compressed and collided along the TSM’s fluid interface, enhancing the transverse diffusion effect during the mixing process [14]. The introduction of grooves also helped in developing chaotic advection and serpentine, proving that grooves are very effective for mixing fluids [15]. A droplet micromixer with zigzag geometry compacted in a short area was developed for efficient mixing at low Re [16]. The passive techniques offer the advantage of ease of development, but flow control within the channel is difficult. Thus, more in-depth research should be done to optimize the existing techniques for better efficiency [17].

Some researchers prefer active methods for generating chaotic advection. For instance, the introduction of alternating electric fields produces electroosmotic vortices that disrupt parallel laminar flow and create chaotic trajectories. Similarly, an orifice-based active micromixer was developed by introducing 3D microelectrodes in the orifices to increase the mixing of fluids [18]. Similarly, Surface Acoustic Waves (SAW) and oscillating bubbles trapped in horseshoe-shaped structures trigger acoustic streaming, which gives rise to severe swirling flows and chaotic advection. Moreover, magnetic actuation can also be useful for this purpose, but the limitation involves the presence of magnetic particles in the fluid to be affected by the magnetic field. An application includes efficient DNA extraction using an on-disk magnetic stirrer [19]. Continuous change in input pressure between the source and sink at the side channels produces a transverse disturbance that results in the generation of chaotic trajectories. Both passive and active methods have their own benefits and limitations. Some researchers are working on the development of hybrid micromixers to produce better results. For example, a hybrid micromixer with micropillars and an acoustic field was developed to produce more efficient results for DNA extraction [20].

Apart from conventional passive and active methods of mixing in microfluidics, some researchers have keenly turned to bioinspired approaches, drawing inspiration from nature’s optimized transport systems to design effective micromixers [21]. Nature has evolved sophisticated mechanisms for fluid transport and mixing across various scales [22]. For example, the vein network of physarum polycephalum provides an intense understanding of how organisms manage the fluid dynamics [23]. It was also highlighted that the flow in Physarum is maximized by contraction patterns adapted to the size of the network. It shows that biological architecture is naturally optimized for mass transport and distribution [24]. Utilizing these biological principles, researchers have developed various bio-inspired mixing techniques. One study shows that active mobility of microorganisms was mimicked to develop a microfluidic system with artificial active cilia. This work was inspired by the beating motion of Paramecia. These artificial cilia were electrically or magnetically operated and helped in inducing chaotic advection at a low Reynolds number [25]. Similarly, an effective microfluidic system was developed, taking inspiration from the blood transport system [26]. Moreover, numerical simulation of a fractal tree-like rectangular network showed its potential in achieving the maximum transport efficiency [27]. Another microfluidic device was developed on the “Split–intersect–recombine principle” inspired by vascular topology. Mitochondria are an important organelle of eukaryotes, also known as the powerhouse of the cell. These are the organelles that can adapt to and respond to various stressors and metabolic demands within the cell. Its dynamics include fission, fusion, transport and mitophagy. Here, optimal functionality is critical for signal transduction and metabolism [28]. Thus, energy is generated in a specific membrane of mitochondria called the cristae. Cristae are multiple tubular invaginations in the matrix separated by cristae junctions. From a functional point of view, the cristae structure was developed into the inner membrane surface, enhancing the capacity of oxidative phosphorylation [29].

In this study, a mitochondria-cristae-inspired matrix was incorporated into a conventional T-channel to examine its effect on fluid mixing. The proposed passive micromixer (“Mito-mixer”) consists of cristae-like baffles and granule-like micropillars, and its mixing performance under laminar flow conditions was numerically evaluated. Three configurations, namely single-, double-, and triple-cristae matrices, were compared with a conventional T-mixer over the range Re=0.1–100 using mixing-index and concentration-based analyses. To evaluate the feasibility of the design architecture, both inlet streams were modeled as water, while the concentration field was treated as a passive scalar under identical fluid properties. This simplified approach is commonly used to assess the geometry-driven mixing capability of a micromixer independently of viscosity-mismatch effects. Since the present numerical framework is based on a water–water system, the proposed design is expected to be particularly relevant in practical applications involving nine low-diffusivity solutes or suspended species in aqueous media, for which diffusion alone is insufficient to ensure rapid mixing in a simple straight microchannel. Such systems may include biomacromolecules, polymer-containing solutions, colloidal suspensions, soft-matter systems, and reactive reagent streams requiring rapid contact under laminar microflow conditions.

Although T-junction and serpentine-based passive micromixers are well established in the literature, the present work explores a morphology-inspired internal architecture that combines repeated cristae-like inset structures with micropillars within a compact T-channel layout. Rather than simply reproducing a conventional serpentine mixer, the proposed geometry is intended to enhance interfacial stretching, flow deflection, and localized flow disturbance over a limited channel length. In addition, the effect of cristae matrix density on mixing performance is systematically examined over a broad Reynolds-number range, thereby clarifying the relationship between geometric complexity and mixing enhancement. Thus, the study provides a numerical assessment of a distinct morphology-inspired passive micromixer architecture based on repeated cristae-like features and micropillar-induced perturbations.

## 2. Methodology

### 2.1. Microchannel Design

Microchannel designs are crucial for efficient fluid mixing. However, bioinspired micromixers extensively depend on their geometry-driven mechanism [26]. In this research, the numerical model was inspired from “mitochondria” by introducing baffles inspired from “cristae” and circular micropillars inspired from “granules” to develop an effective mitochondrial-inspired micromixer, the “Mito-mixer”. The combination of baffles and micropillars can be utilized to produce chaotic advection for mixing. This novel bio-inspired matrix design was incorporated into conventional T-type micromixers to evaluate the effectiveness of this geometry.

Thus, to design the Mito-mixer, each geometric parameter was explicitly defined to generate laminar mixing regimes. Low-aspect-ratio cross-sections were favorable for generating Dean vortices as compared to high-aspect-ratio cross-sections [30]. A channel height of 50 µm was selected as an optimal value for fabrication using SU-8 photoresist (MicroChem Corp., Westborough, MA, USA) and PDMS soft lithography (Sylgard 184, Dow Corning, Midland, MI, USA), ensuring reliable pattern definition and structural integrity. Henceforth, a height of 50 µm with a width of 200 µm (AR = 0.25) may provide a balance between the desired application and manufacturing considerations. All the parameters for designing a Mito-mixer are listed in Table 1 below.

**Figure 1:**
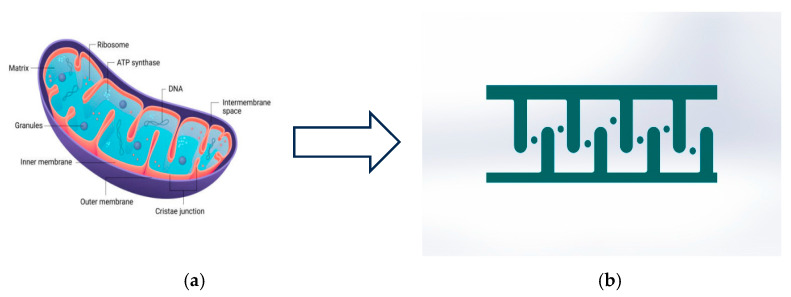
(**a**) Structure of mitochondria [29]. (**b**) Unit matrix of microchannel inspired by mitochondria.

The cristae throat constriction (W_2_ = 60 µm; W_2_/W_1_ = 0.3) was designed to accelerate the flow by continuity. This implication increased the local velocity of the fluid at the throat by 3.33 times the initial flow velocity. A local inertial spike increased the effective Reynolds number and assisted in generating vortices using a nozzle-diffuser effect. Then, the baffle pitch (P = 150 µm ≈ 2 Dₕ) regularly regenerated these secondary flows with spacing that supported vortex development before viscous decay. And finally, the rounded cristae curvature (R ≈ 30 µm) elevated the Dean number ($De=Re\sqrt{D_{h}/(2R_{c})}$), and escalated curvature-driven secondary vortices while avoiding sharp corners, which can hurt residence-time uniformity. A 30 µm pillar placed in the turn (blockage ratio ≈ 0.15) disturbed the core flow with minimal pressure drop by splitting slow-mixing streamlines and pushing fluid into high-shear regions. Its laminar wake ($Re_{D}=\rho V_{\mathrm{local}}D/\mu$) may synergize with centrifugal effects to amplify transverse transport. Overall, geometry was selected to regulate Re/De and to control disturbances, thereby enabling predictable secondary flows and improved mixing (Figure 1).

**Figure 2:**
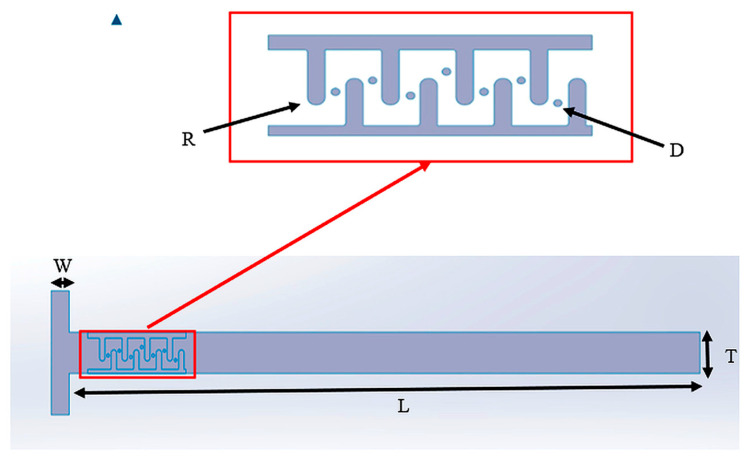
Schematic of the proposed mitochondria-inspired micromixer geometry.

Using the previously mentioned parameters, the micromixer geometry was modeled in SolidWorks (SW 2025) (Figure 2).

**Figure 3:**
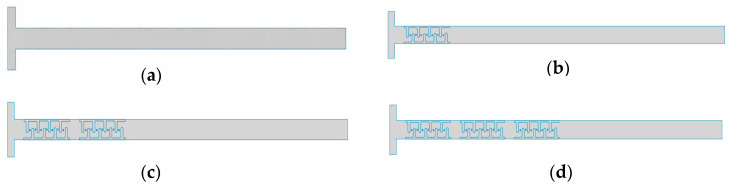
The geometries used for analysis: (**a**) T-channel, (**b**) T-channel with single crista, (**c**) T-channel with double cristae, and (**d**) T-channel with triple cristae.

Four similar geometries with different numbers of cristae matrices induced in the T-channel were designed for numerical simulations using COMSOL Multiphysics (6.1 version) (Figure 3). Different geometries in the T-channel were investigated in 2D in order to observe how the fluid behaves and how it affects the mixing of fluids.

**Figure 4:**
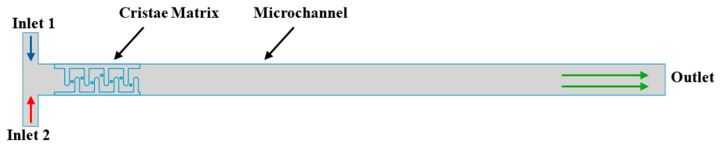
Schematic of the computational domain for the 2D simulation of the T-channel.

In the simulation, the fluid enters the microchannel from opposite sides of the T-shaped channel through two opposing inlets. It then passes through the cristae matrix into the main channel, which serves as the primary region of interest for observing fluid behavior (Figure 4).

### 2.2. Governing Equations and Numerical Methodology

The fluid flow and species transport in the proposed micromixer were modeled numerically in COMSOL Multiphysics using two coupled physics interfaces: Laminar Flow and Transport of Diluted Species. Since the working fluid was water and the investigated Reynolds-number range corresponds primarily to laminar microchannel flow, the flow was assumed to be steady, incompressible, Newtonian, and isothermal. Under these assumptions, the governing equations are the continuity equation, the Navier–Stokes equations for momentum conservation, and the convection–diffusion equation for species transport.

#### 2.2.1. Continuity and Momentum Conservation

For incompressible flow, mass conservation is expressed as [34]:

(1) $$\nabla \cdot \vec{u}=0$$

The steady Navier–Stokes equation is [14]:

(2) $$\rho \left(\vec{u}\cdot \nabla \right)\vec{u}=-\nabla p+\mu \nabla^{2}\vec{u}+\vec{F}$$

where $\vec{u}$ is the velocity vector, ρ is the fluid density, p is the pressure, μ is the dynamic viscosity, and $\vec{F}$ represents any external body force. In the present study, no additional body force was imposed, so $\vec{F}=0$.

#### 2.2.2. Laminar Flow (spf)

The Laminar Flow interface in COMSOL was used to solve Equations (1) and (2) throughout the computational domain. This interface is appropriate for low-Reynolds-number microfluidic flow, where viscous effects dominate and turbulence is absent. Although creeping-flow conditions may be used at extremely low Reynolds numbers, the laminar-flow model was adopted here to maintain a consistent formulation over the entire investigated range of Re = 0.1 to 100.

#### 2.2.3. Transport-Diluted Species Model

To evaluate mixing between the two inlet streams, the Transport of Diluted Species interface was coupled with the velocity field obtained from the laminar-flow solution. The transport of species was governed by the steady convection–diffusion equation [35]:

(3) $$\vec{u}\cdot \nabla c=D\nabla^{2}c$$

where *c* is the concentration of species, and *D* is the diffusion coefficient.

#### 2.2.4. Brief Numerical Methodology

The geometry of the T-shaped micromixer and the cristae-based configurations was constructed in SolidWorks and imported into COMSOL Multiphysics for simulation. A stationary solver was employed to obtain steady-state flow and concentration fields. First, the laminar velocity and pressure fields were computed, and these were then coupled to the species transport model to solve for concentration distribution. The two inlet streams were assigned different concentrations, allowing the degree of mixing to be quantified at the outlet and along the channel length using the mixing index.

### 2.3. Boundary Conditions

In this research, water was selected as the material’s domain, and it was inserted into the inlet through channel 2. For our observation, the concentration at inlet 2 was considered as “1” and consequently at inlet 1 as “0”. The inlet velocity of both inlets (1 and 2) may be expressed as a function of Reynolds number (Re):

(4) $$U_{in}=\frac{Re\times \mu }{\rho \times D_{h}}$$

where *U_in_* is the inlet velocity.

The outlet pressure was set to P_0_ = 0 Pa. Initial values for computations were set to zero by default. Parametric sweeps were applied in each geometry to compute the results at different Reynolds numbers: 0.1, 1, 10, 25, 50 and 100. Multiple results were obtained using different inlet velocities. The water and hydraulic diameters were 80 µm, as determined from the earlier discussion. The properties of water are listed in Table 2.

The Péclet number puts convective and diffusive transport phenomena in correlation [34]. Hence, the Péclet number was evaluated to characterize the relative importance of convective and diffusive transport in the present micromixer. Pe = Re·Sc [35] was calculated for each Re and indicated in Table 3 below.

### 2.4. Performance Evaluation

The objective of this research was to observe the mixing of the fluids in the novel bio-inspired microchannel at different Reynolds numbers. Typically, in microfluidics, two fluids enter at different concentrations (e.g., dye versus pure solvent). Since the flow is laminar, mixing occurs mainly by diffusion and interface stretching, not by turbulence. The Mixing Index (MI) is a quantitative measure of the degree of uniformity in concentration at a given cross-section, used to evaluate the effectiveness of the design, and may be calculated by the formula [20]:

(5) $$MI=1-\sqrt{\frac{\sigma^{2}}{\sigma_{max}^{2}}}$$

(6) $$\sigma =\sqrt{\frac{1}{n}\sum_{i=1}^{n}\left(c_{i}-\bar{c}\right)}$$

(7) $$\sigma_{max}=\sqrt{\bar{c}(1-\bar{c})}$$

where *c_i_*—local concentration, $\bar{c}$—mean concentration, σ—standard deviation of concentration, and *σ_max_*—maximum possible standard deviation. The mixing indices were evaluated at the outlet of the geometry for each case. The mean concentration $\bar{c}$ was obtained using a line average across the outlet, while local concentrations *c_i_* were extracted at different outlet heights using point evaluation. These values of $\bar{c}$ and *c_i_* were then used to evaluate the corresponding mixing indices (MI).

In addition to the outlet mixing index, the mixing index was calculated at multiple points along the length to assess the effectiveness of the geometry.

### 2.5. Grid Independence

In numerical investigations, a balance between accuracy and computational resources is crucial for effective study. Here, the mesh-independence test was applied to a single study. The mesh providing the lowest discretization error with the minimum number of elements was selected for the entire study. To assess mesh independence, the microchannel with two matrices of cristae was tested at Re = 0.1 on different meshes. The generated mesh comprised tetrahedral elements at the sides of the channel and triangular elements in the rest of the geometry (Table 4). It may be observed that after level 5, the results are almost the same.

**Figure 5:**
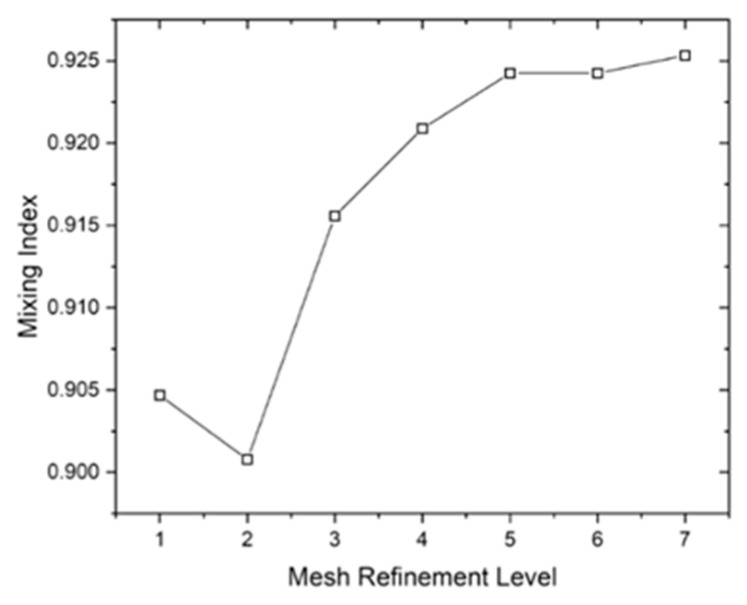
Grid independence test.

The grid independence test (Figure 5) showed that the simulation results became stable as the mesh was refined. After a certain point, further increasing the mesh density caused only very small changes in the results, indicating that the solution no longer depends on the mesh size and is therefore reliable.

**Figure 6:**
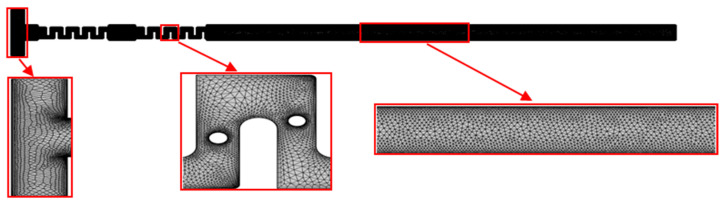
Level 5 “Finer” mesh of T-channel with two matrices of cristae.

Thus, for further numerical studies, a level 5 “Finer” mesh was used for the T-channel with two matrices of cristae (Figure 6).

### 2.6. Model Validation

**Figure 7:**
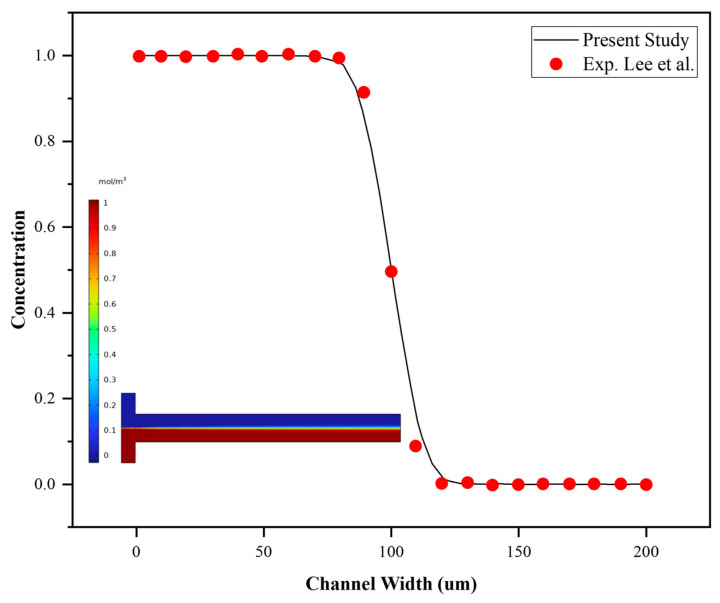
Validation of the present numerical model against the experimental data of Lee et al. for a conventional T-shaped micromixer without barriers. The normalized concentration profile across the channel width at x = 1 cm downstream under Re = 8 shows good agreement between the present simulation and the published experimental data digitized from Lee et al. [36].

To validate the numerical framework, a conventional T-shaped micromixer reported by Lee et al. [36] was reproduced under the same operating conditions of Re = 8. The normalized concentration profile across the channel width was extracted at a downstream distance of 1 cm from the T-junction and compared with the published experimental profile. As shown in Figure 7, the present numerical prediction agreed well with the experimental data, particularly in terms of the interface location, concentration transition, and plateau regions. This agreement confirmed that the adopted numerical setup is capable of accurately capturing convection–diffusion behavior in a standard micromixer configuration.

Although 3D simulations provide more physically realistic results, 2D simulations can still yield reasonably accurate predictions while significantly reducing computational costs, even for relatively low aspect ratios of about 0.18 (height-to-width ratio) [35].

## 3. Results and Discussion

### 3.1. Qualitative Flow Topology and Concentration Distribution

**Figure 8:**
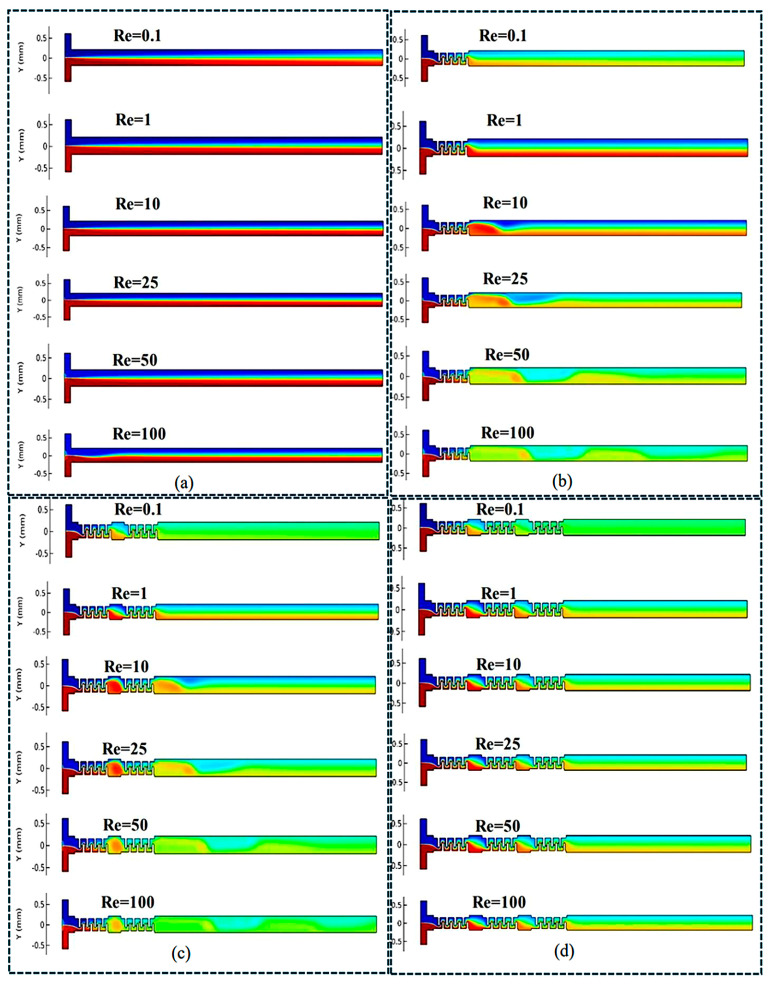
Concentration distribution across different micromixer configurations (Red = 1 and Blue = 0; Range = 0–1): (**a**) T-channel with Re = 0.1–100; (**b**) T-channel with a single matrix with Re = 0.1–100; (**c**) T-channel with a double matrix with Re = 0.1–100; (**d**) T-channel with a triple matrix with Re = 0.1–100.

The mixing performance of the proposed Mito-mixer was first analyzed through qualitative analysis of concentration distributions at the channel outlet for Reynolds numbers spanning between 0.1 and 100 (Figure 8). The obtained results compare the concentration fields at Re ranging from 0.1 to 10 with different configurations of geometries (T-channel, and T-channels with single crista, double cristae and triple cristae). Results showed that a little diffusion can be seen in the T-channel at Re = 0.1, but after that, the concentration remained similar at Re = 1, 10, 25, 50 and 50. It indicated that when the Re number increases, the diffusion-dominated mixing process diminishes in a simple T-channel.

On the contrary, the cristae-equipped geometries introduced repeated disturbances in the flow, generating localized circulation. This behavior was consistently observed across all cristae-designed T-channel geometries, indicating that those cristae matrices systematically enhance interfacial deformation and transverse transport. As a result, the overall mixing performance was highly improved in these configurations. At low Reynolds numbers (Re = 0.1–1), diffusion was still significant due to long residence time. However, the presence of the cristae structure resulted in earlier interface stretching relative to the base model. At the intermediate Reynolds number range (Re = 10–25), the concentration interface became noticeably distorted, indicating a transition in which advective transport begins to compete with diffusion. At high Reynolds number range (Re = 50–100), the chaotic advection became prominent, and the concentration field approached a more uniform distribution. Overall, the results of numerical simulations (Figure 8) confirmed that the introduction of cristae-structure matrix into the T-channel improves mixing by repeatedly stretching, folding, and laterally transporting fluids relative to their baseline. Moreover, increasing the density of cristae elements in the channel enhances the mixing of elements and leads to a better uniform concentration distribution at the outlet.

### 3.2. Effect of Reynolds Number on Mixing Efficiency

**Figure 9:**
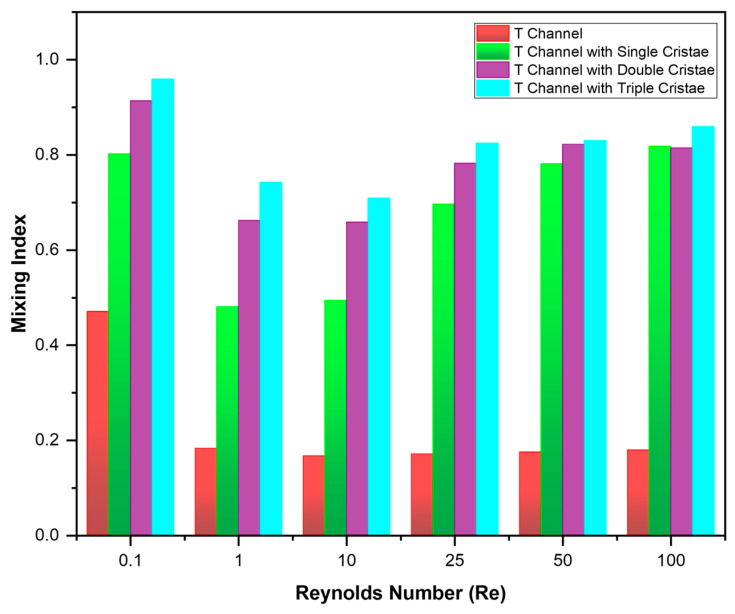
Mixing indices at different Reynolds numbers.

The influence of Reynolds’ number on mixing efficiency was quantified using the mixing index (MI) evaluated at the outlet. A key feature of the results was the non-monotonic dependence of MI on Reynolds number for the cristae-based designs (Figure 9). It was observed that MI is relatively high at the lowest Reynolds number Re = 0.1, where diffusion is prominent due to high residence time. After, MI decreases significantly in the low-Reynolds number range (Re = 1–10), where the residence time is short, reducing the time available for diffusion. Apart from the short diffusion time, the flow is not strong enough to generate substantial chaotic advection. Although MI is improving with respect to cristae density at this level as well, the results remain typical at these flow rates. As the Reynolds number increases to higher values (Re ≥ 25), the mixing efficiency improves significantly, indicating that the cristae enhance chaotic advection within the flow regime.

The mixing index as a function of the Reynolds number (Re) for four different micromixer geometries (Figure 9) showed distinct trends, highlighting the influence of channel design on mixing performance across different flow regimes. As shown in Table 5, the T-channel exhibits some diffusion at Re = 0.1; however, beyond this point, the mixing index (MI) remains nearly constant at a low level—approximately 47% at Re = 0.1 and around 16–18% for Re = 1–100. The T-channel with single crista had a MI of 80.13% at Re = 0.1, then decreased to 48–49% for Re = 1–10 and again started increasing for Re = 25–100 with MI values of 69.55%, 78.08% and 81.7% at Re = 25, 50 and 100, respectively. Similar behavior was observed in the T-channel with double cristae and triple cristae with maximum MI values of 91.33% and 95.85% respectively, at Re = 0.1. Then, MI decreased for both at Re = 1–10, with MI values of 65–66% and 70–74%, and achieving the maximum MI of 81.5% and 85.8% at Re = 100, respectively. Thus, the maximum MI was achieved in the T-channel with triple-cristae matrices of 95.86%, 74.17%, 70.85%, 82.41%, 82.92%, and 85.85% at Reynolds numbers of 0.1, 1, 10, 25, 50, and 100, respectively. It is evident that the cristae-equipped configurations consistently achieve higher MI than the plain T-geometry, confirming enhanced mixing. Increasing cristae matrix density further improves performance, with the densest configuration showing the highest MI in most cases. The difference is most prominent in the intermediate Reynolds-number range (Re = 1–25), where the fluids constantly require the folding and stretching mechanism for mixing. At higher Reynolds numbers (Re = 50–100), the MI of single, double and triple cristae matrices is comparable, but still increases with the number of cristae. At Re = 100, the single-cristae geometry can yield a higher MI than the double-cristae case because the added structures may promote preferential high-velocity paths and reduce effective residence time in the mixing region. In addition, the secondary flows generated by the double-cristae arrangement can partially counteract interfacial stretching, resulting in lower outlet uniformity for this specific regime.

### 3.3. Spatial Evolution of Mixing Along the Channel

**Figure 10:**
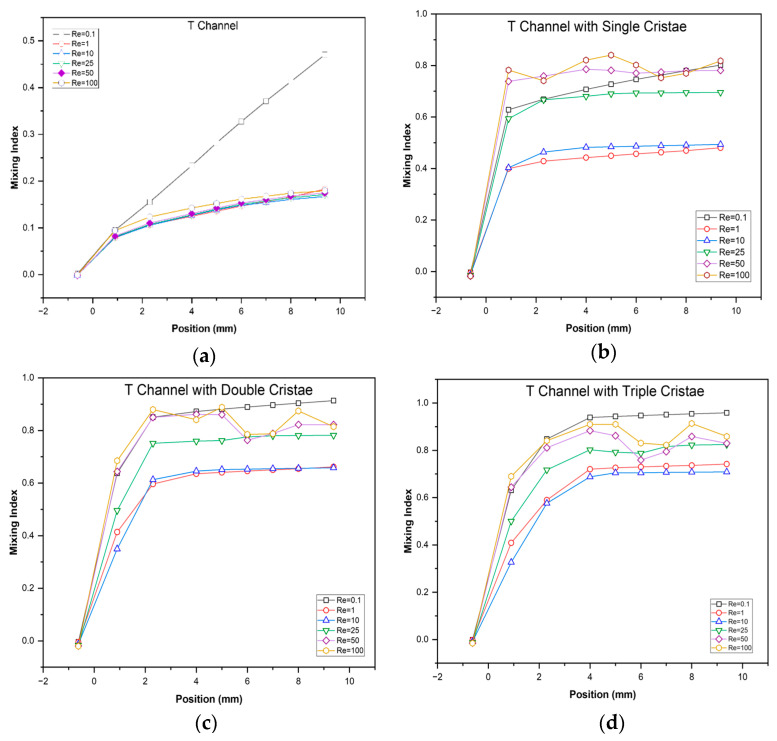
Spatial evolution of mixing indices along the (**a**) T-channel and (**b**) T-channel with single, (**c**) double and (**d**) triple cristae.

To demonstrate the effectiveness of geometry, the spatial evolution of mixing along the channel was investigated. Rapid mixing along the channel indicates a more effective and length-efficient design. For this purpose, MI was observed at multiple points along the channel for each geometry at Reynolds numbers (Re) ranging from 0.1 to 100 (Figure 10).

The results (Figure 10) showed the axial evolution of MI in different geometries at different Reynolds numbers (Re = 0.1–100). The T-channel showed a gradual change in MI along the length, indicating that slow diffusion is taking place along the length. Diffusion was better at low Re = 0.1, but it almost diminished at higher Reynolds numbers. Thus, T-channels with single, double or triple cristae matrices showed a significant increase in MI. T-channels with two and three cristae matrices showed better MI than a single-cristae matrix but required longer length to gain uniformity due to high chaotic advection at high Reynolds numbers ranging from 50 to 100. So, increased cristae density improved both the mixing rate and the final mixing uniformity, making the design more efficient.

### 3.4. Pressure Drop and Figure of Merit Analysis

In addition to mixing efficiency, the hydraulic resistance of the proposed micromixer must also be considered to evaluate its practical feasibility. Therefore, the pressure drop across the single-, double-, and triple-cristae configurations was analyzed over the investigated Reynolds-number range, and a figure of merit (FoM) was introduced as FoM = MI/∆P [20], where MI is the mixing index, and ∆P is the pressure drop along the channel. The results showed that the pressure penalty increased with Reynolds number for all configurations, while the FoM decreased significantly as the flow rate increased. Among the tested cases, the single-cristae configuration exhibited the highest FoM throughout the investigated range, followed by the double- and triple-cristae designs (Table 6).

For example, the FoM values at Re = 0.1 were 7.945, 4.535, and 3.178 mPa^−1^ for the single-, double-, and triple-cristae configurations, respectively, whereas at Re = 100, these values decreased to 0.00129, 0.000664, and 0.000464 mPa^−1^. This indicates that although increasing cristae density improves the mixing index, it also imposes a larger hydraulic penalty. Hence, the denser cristae configurations are advantageous when high mixing performance is the main objective, while the single-cristae design provides a better balance between mixing enhancement and pressure-drop cost.

**Figure 11:**
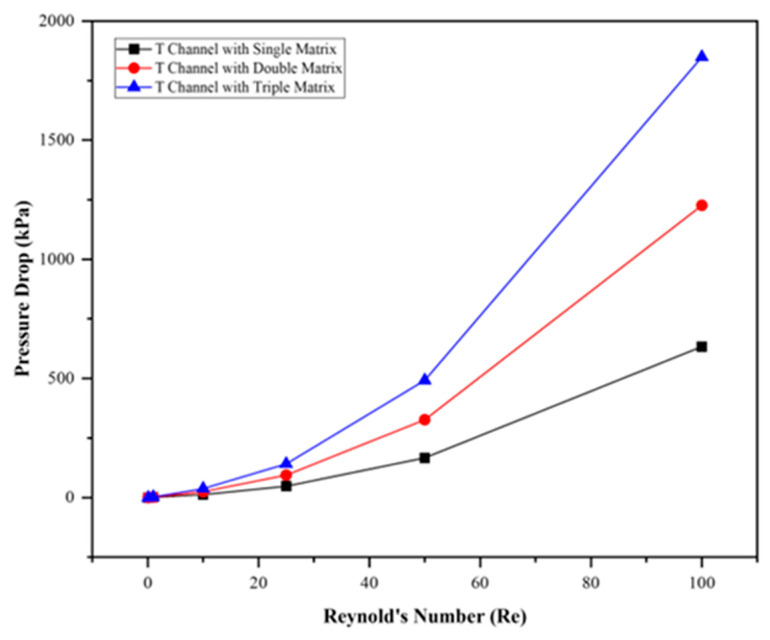
Pressure drops for each configuration.

Further simulation results showed that the pressure drop increased markedly with increasing Reynolds number for all micromixer configurations, reflecting the greater hydraulic resistance associated with higher flow velocities. Figure 11 indicates that enhanced mixing is achieved at the expense of increased pumping power, highlighting the inherent trade-off between mixing performance and hydraulic cost in the proposed designs.

### 3.5. Evaluation at a Narrow Channel Widths

**Figure 12:**
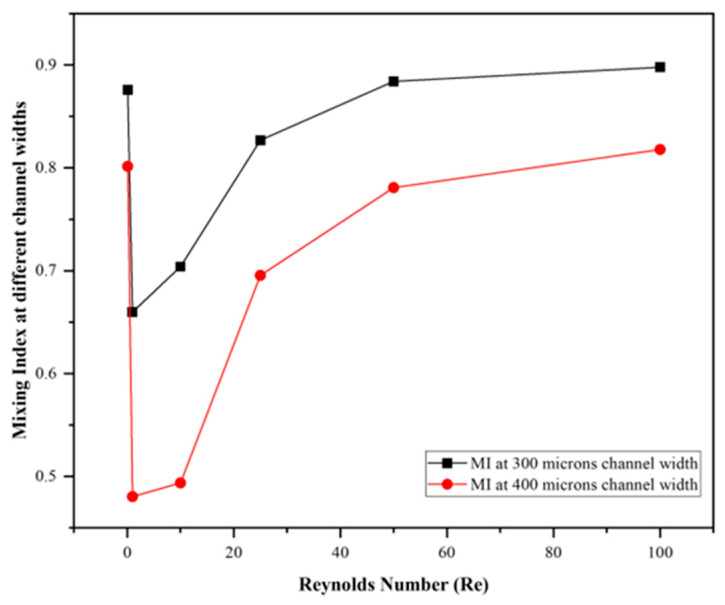
Comparison of mixing indices at different channel widths (300 and 400 microns) for the T-channel with single crista.

To examine the influence of the main channel width, an additional comparison was carried out between 0.3 mm and 0.4 mm channel widths for the single-cristae configuration (Figure 12). The narrower 0.3 mm channel consistently produced higher mixing indices at the monitored downstream sections, indicating that stronger confinement promotes enhanced interfacial stretching and mixing. For example, at the 400 µm section, the mixing index at Re = 25, 50, and 100 increased from 0.6955, 0.7808, and 0.8179 to 0.8269, 0.8840, and 0.8978, respectively, when the width was reduced from 0.4 mm to 0.3 mm. Table 7 further shows that the narrower channel generally imposed a higher pressure drop over most of the investigated Reynolds-number range, confirming the hydraulic penalty associated with increased confinement. Although the 0.4 mm case showed a slightly higher pressure drop at Re = 100, the overall trend indicates a clear trade-off between improved mixing and increased hydraulic resistance. Therefore, the selected 0.4 mm width was retained as a more balanced design choice for the present study.

Moreover, a comparison of the pressure drop for different channel widths of the T-channel with single crista was performed (Table 7). The results showed that the pressure drop at a narrower width is greater than at a wider width, and this effect will increase as the cristae density increases.

### 3.6. Comparison of Mixing Index with Some Bio-Inspired Geometries

To evaluate the effectiveness of the proposed mitochondria-inspired micromixer, its performance was compared with several previously reported bio-inspired geometries operating over similar Reynolds number ranges. Some bio-inspired designs, such as pine-tree structures, nature-inspired mini-channel patterns or blood-transport analogs, were widely explored to enhance passive mixing in laminar microflows by promoting interfacial stretching, flow splitting, and secondary circulation (Table 8).

Table 8 shows the mixing performance of selected bio-inspired micromixers with the proposed mitochondria-inspired (cristae-based) design across relevant Reynolds number ranges. The pine-tree-inspired mixer [35] reported very high efficiencies at Re = 10–25, but showed a notable drop at higher Re, whereas the novel T-channel triple-cristae design maintained high outlet MI at Re ≥ 25 with fewer repeated structural units. In the low Re regime, this new design exceeded the nature-inspired mini-channel mixer [21] at Re = 0.1 and provided comparable-to-improved performance depending on cristae density at Re = 1. Compared with the blood-transport-inspired micromixer [26], this new design delivered much higher MI at Re = 0.1 for all cristae configurations, i.e., with the triple-cristae matrix showing the greatest improvement.

### 3.7. Future Experimental Framework

**Figure 13:**
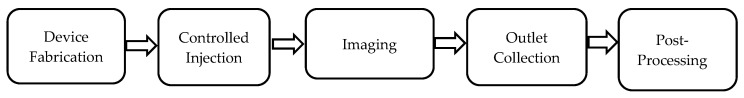
The workflow of experimental procedure of Mito-mixer design.

Based on the numerical results obtained in this research, an experimental framework was proposed to validate mixing performance in the T-channel type Mito-mixer (Figure 13).

The setup was based on a dual-channel syringe pump to deliver two input streams at prescribed flow rates, a PDMS (or clear polymer) microfluidic chip containing the proposed triple-cristae microchannel, microbore tubing and connectors, an inlet fluid reservoir, an optical microscope coupled with a high-resolution camera, and a computer workstation for the real-time visualization and recording of concentration fields and flow features.

**Figure 14:**
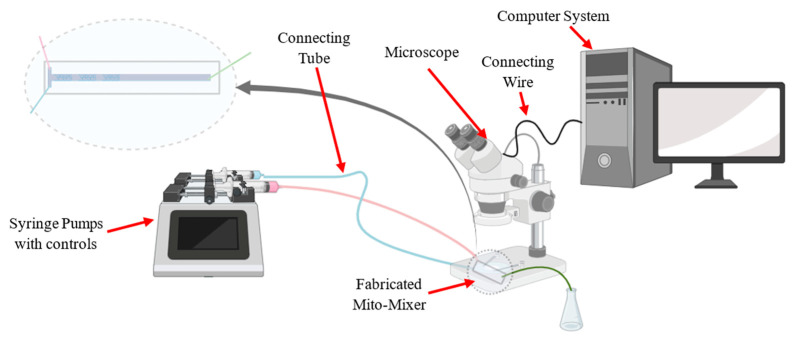
Proposed experimental framework for future investigations.

For the fabrication of microchannels (Figure 14), the master pattern may be produced using a high-resolution SLA 3D printer (e.g., clear photopolymer resin) or a micromachined/printed mold, followed by PDMS casting and bonding to a glass slide to obtain a transparent device suitable for microscopy. The triple-cristae matrix may be implemented as a regular, repeatable internal microstructure to investigate its influence on interface stretching and folding, particle transportation, and stability during mixing. The fluids with two deionized water streams dyed with different color tracers may be used for flow visualization and mixing observations. Thus, image acquisition may be performed at selected downstream locations (including the outlet plane) and processed to compute mixing index, concentration profiles, and spatial evolution of mixing, enabling a direct comparison with the numerical results.

## 4. Discussion

The present numerical investigation demonstrated that the proposed cristae-based Mito-mixer provides a clear improvement over the conventional T-channel in terms of passive mixing performance. While the basic T-channel remains largely diffusion-limited, the cristae-equipped configurations introduce repeated interface deformation and transverse redistribution, promoting more uniform concentration fields along the channel and at the outlet. These observations confirmed that the proposed morphology-inspired geometry enhances mixing through internal structural modification alone, without the need for external actuation, which is a key advantage for compact passive microfluidic systems.

A notable outcome of the study was the non-monotonic dependence of the mixing index on Reynolds number. At very low Reynolds numbers, the mixing index remained relatively high because the long residence time allowed diffusion to act effectively. As Reynolds number increased into the intermediate range, the available diffusion time decreased, while the advective disturbances were still not sufficiently strong to produce fully developed geometric mixing. This led to a reduction in the mixing index. At higher Reynolds numbers, however, the cristae structures became increasingly effective in promoting interfacial stretching, lateral transport, and secondary-flow development, which resulted in a renewed increase in mixing performance. This behavior indicates that different transport mechanisms dominate in different flow regimes, and that the proposed design remains effective over a broad Reynolds-number range.

The results further showed that cristae density plays an important role in determining performance. In general, increasing the number of cristae matrices improved outlet concentration uniformity and accelerated the spatial development of the mixing index along the channel. Higher-density cristae configurations therefore offer an advantage when compact and length-efficient mixing is required. At the same time, the pressure-drop analysis revealed that the improved mixing was accompanied by increased hydraulic resistance. The figure-of-merit results indicated that lower-density configurations provide a more favorable balance between mixing enhancement and hydraulic cost, whereas higher-density configurations are preferable when maximum outlet mixing is the primary design objective. This highlighted that the most suitable geometry depends on the practical requirements of the intended application.

The additional comparison between 0.3 mm and 0.4 mm channel widths further supported this interpretation. The narrower channel showed improved mixing because stronger confinement promoted greater interfacial stretching and transverse transport. However, this gain was generally associated with a higher pressure-drop penalty, confirming that channel narrowing improves mixing at the cost of hydraulic efficiency. Thus, the selected 0.4 mm width may be regarded as a balanced design choice rather than a geometry aimed solely at maximizing the mixing index.

From an application perspective, the proposed Mito-mixer is particularly relevant for passive microfluidic systems in which external forcing is undesirable, and rapid homogenization must be achieved within a limited channel length. This includes lab-on-a-chip devices, biochemical reagent handling, microscale reaction initiation, and aqueous systems involving relatively low-diffusivity solutes or suspended species. In such cases, such a geometry-driven micromixer may provide a practical alternative to conventional T- and Y-type mixers that rely predominantly on slow molecular diffusion. The comparison with selected bio-inspired geometries also indicated that the proposed design remained competitive across relevant Reynolds-number ranges, particularly in terms of compact mixing performance and outlet uniformity.

Although the present study established the baseline performance and practical benefits of the proposed architecture, some aspects remain open for future investigation. In particular, the transport analysis was carried out using a single representative diffusion coefficient for an aqueous scalar field, and the specific role of the granule-inspired micropillars was not isolated through a separate with/without-pillar comparison. These extensions would provide a broader assessment of applicability and would further clarify the contribution of the individual design elements.

## 5. Conclusions

This study numerically investigated a mitochondria-inspired passive micromixer incorporating cristae-like baffles and granule-like micropillars into a conventional T-channel. Compared with the basic T-channel, the cristae-based configurations produced stronger interface deformation, improved transverse redistribution, and more homogeneous concentration fields along the channel and at the outlet. The results showed that the mixing index depends strongly on Reynolds number and generally improves with increasing cristae density, particularly at higher Reynolds numbers where geometry-induced advection becomes more effective.

The study also demonstrated that enhanced mixing is achieved at the expense of increased hydraulic resistance. Therefore, a clear trade-off exists between mixing performance and pressure-drop penalty, and the preferred cristae density should be selected according to application requirements. Overall, the proposed Mito-mixer provides a promising passive-mixing strategy for compact microfluidic systems requiring improved mixing over a broad Reynolds-number range.

## Figures and Tables

**Table 1:** Geometric parameters and design specifications.

Parameter	Symbol	Value	Fluid Mechanical Role (Theory)	Key Reference
Channel Length	L	10 mm	Industry standard cutoff for efficiency	-
Hydraulic Dia.	D_h_	80 μm	Determines Laminar/Transitional Regime limit.	[30,31,32]
Constriction	W_2_/W_1_	0.3	Induces flow separation and jetting (Bernoulli).	[9]
Curvature	R	30 μm	Maximizes Dean Vortices (Secondary flows). Equation: De∝R^−0.5^	[32]
Obstruction	D	30 μm	Splits streamlines and wake generation.	[33]
Inlet Width	W	200 μm	-----	
Main Channel Width	T	400 μm	-----	

**Table 2:** Material properties of water.

Property	Value	Reference
Density (ρ)	1000 kg/m^3^	[34]
Dynamic Viscosity (µ)	10^−3^ Pa·s	[34]
Diffusion Coefficient (D_c_)	10^−9^ m^2^·s^−1^	Used in COMSOL Simulation

**Table 3:** Calculation of Péclet number across different Re.

Reynolds Number (Re)	Schmidt Number (Sc)	Péclet Number (Pe)
0.1	1000	100
1	1000	1000
10	1000	10,000
25	1000	25,000
50	1000	50,000
100	1000	100,000

**Table 4:** Results of the mesh independence test.

Sr. #	Mesh Type	Number of Elements	Number of Boundary Elements	Mixing Index
1	Coarser	12,429	1373	0.904679
2	Coarse	21,506	1926	0.900785
3	Normal	25,046	2066	0.91557
4	Fine	36,044	2516	0.920891
5	Finer	53,082	3156	0.924248
6	Extra Fine	102,449	4673	0.924248
7	Extremely Fine	117,189	4673	0.925329

**Table 5:** Mixing indices at different Reynolds numbers.

Re	Mixing Index (MI)			
	T-Channel	T-Channel with Single Crista	T-Channel with Double Cristae	T-Channel with Triple Cristae
0.1	0.470413	0.801377	0.913349	0.958558
1	0.182531	0.480433	0.661731	0.741707
10	0.166882	0.493697	0.658265	0.708524
25	0.170925	0.695533	0.781945	0.824138
50	0.174378	0.780812	0.822019	0.829245
100	0.179153	0.817895	0.814494	0.858444

**Table 6:** Figure of Merit (FoM) at every Re for each configuration.

Re	FoM = MI/∆P (mPa^−1^)		
	T-Channel with Single Crista	T-Channel with Double Cristae	T-Channel with Triple Cristae
0.1	7.945097	4.53513	3.178092
1	0.473779	0.326806	0.244598
10	0.038937	0.026056	0.018731
25	0.014583	0.008283	0.005822
50	0.004678	0.002516	0.001684
100	0.001291	0.000664	0.000464

**Table 7:** Comparison of the pressure drop for different channel widths of the T-channel with single crista.

Re	Pressure Drop at 300 Microns (kPa)	Pressure Drop at 400 Microns (kPa)
0.1	0.110714	0.100864
1	1.114943	1.014044
10	14.37551	12.67931
25	53.82279	47.69376
50	168.2673	166.9268
100	578.0935	633.6718

**Table 8:** Comparison of the recent research related to bio-inspired microchannel geometries.

Author	Design Feature	Reynolds Number	Reported Mixing Performance	Comparison with the Current Study
[35]	Pine tree-inspired micromixer	10–100	MI ≈ 99% (Re = 10), 87% (Re = 25), 70% (Re = 50), 44% (Re = 100)	Present study (triple-cristae): MI = 82.41% (Re = 25) and 85.84% (Re = 100), achieving high mixing with fewer repeated units (cristae matrices) than the extensively repeated triangular matrices reported.
[21]	Nature-inspired mini-channel mixer	0.1–1	MI ≈ 73% (Re = 0.1), 78% (Re = 1)	Present study: MI = 80.14–95.86% at Re = 0.1 (single–triple) and 48.04–74.17% at Re = 1 (single–triple).
[26]	Blood-transport-inspired micromixer	0.01–0.1	MI ≈ 79% (Re 0.1)	At Re = 0.1, the present study exceeds the reported value for all cristae cases: 80.14% (single), 91.33% (double), 95.86% (triple).

## Data Availability

The original contributions presented in the study are included in the article; further inquiries can be directed to the corresponding author.
